# Parallel evolution of TCP and B-class genes in Commelinaceae flower bilateral symmetry

**DOI:** 10.1186/2041-9139-3-6

**Published:** 2012-03-06

**Authors:** Jill C Preston, Lena C Hileman

**Affiliations:** 1 Department of Ecology and Evolutionary Biology, University of Kansas,1200 Sunnyside Avenue, Lawrence, KS 66045, USA

**Keywords:** B class genes, Commelinaceae, *CYCLOIDEA*, homeotic change, monocots, tepals, *teosinte branched1*

## Abstract

**Background:**

Flower bilateral symmetry (zygomorphy) has evolved multiple times independently across angiosperms and is correlated with increased pollinator specialization and speciation rates. Functional and expression analyses in distantly related core eudicots and monocots implicate independent recruitment of class II TCP genes in the evolution of flower bilateral symmetry. Furthermore, available evidence suggests that monocot flower bilateral symmetry might also have evolved through changes in B-class homeotic MADS-box gene function.

**Methods:**

In order to test the non-exclusive hypotheses that changes in TCP and B-class gene developmental function underlie flower symmetry evolution in the monocot family Commelinaceae, we compared expression patterns of *teosinte branched1 *(*TB1*)-like, *DEFICIENS *(*DEF*)-like, and *GLOBOSA *(*GLO*)-like genes in morphologically distinct bilaterally symmetrical flowers of *Commelina communis *and *Commelina dianthifolia*, and radially symmetrical flowers of *Tradescantia pallida*.

**Results:**

Expression data demonstrate that *TB1*-like genes are asymmetrically expressed in tepals of bilaterally symmetrical *Commelina*, but not radially symmetrical *Tradescantia*, flowers. Furthermore, *DEF*-like genes are expressed in showy inner tepals, staminodes and stamens of all three species, but not in the distinct outer tepal-like ventral inner tepals of *C. communis*.

**Conclusions:**

Together with other studies, these data suggest parallel recruitment of *TB1*-like genes in the independent evolution of flower bilateral symmetry at early stages of *Commelina *flower development, and the later stage homeotic transformation of *C. communis *inner tepals into outer tepals through the loss of *DEF*-like gene expression.

## Background

Evolutionary transitions between flower radial symmetry (polysymmetry and actinomorphy) and bilateral symmetry (monosymmetry and zygomorphy) have occurred multiple times independently across angiosperms, and are associated with increased pollinator specialization and speciation rates [1-6]. Indeed, some of the largest angiosperm families have species with predominantly bilaterally symmetrical flowers, including the legumes (Leguminosae, rosids, core eudicots), daisies (Asteraceae, asterids, core eudicots) and orchids (Orchidaceae, monocots) [7]. Recent functional studies in distantly related core eudicots - including *Antirrhinum majus *(Plantaginaceae), *Iberis amara *(Brassicaceae), *Pisum sativum *and *Lotus japonicus *(Leguminosae) and *Gerbera hybrida *(Asteraceae) - have demonstrated a role for class II TEOSINTE BRANCHED1 (TB1)/CYCLOIDEA (CYC)/PROLIFERATING CELL NUCLEAR ANTIGEN GENE-CONTROLLING ELEMENT BINDING FACTOR (PCF) (TCP) transcription factors in establishing flower symmetry by specifying identity to the dorsal (adaxial) region of the flower [8-13]. Since bilateral symmetry has evolved independently in these lineages, genetic data suggest parallel recruitment of class II TCP genes in the evolution of a convergent trait [11,14-16]. Whether homologous TCP genes have been similarly utilized in monocot flower bilateral symmetry remains largely untested [but see [17,18]].

The genetic basis of flower bilateral symmetry is best understood in the model species *A. majus*, and involves the action of four asymmetrically expressed transcription factors [8,9,19-21]. Dorsal identity is specified by the class II TCP genes *CYCLOIDEA *(*CYC*) and *DICHOTOMA *(*DICH*), and the MYB gene *RADIALIS *(*RAD*), whereas ventral (abaxial) identity is conferred by the MYB gene *DICHOTOMA *(*DICH*). *CYC *and *DICH *are derived from a recent duplication event at the base of the Antirrhineae tribe [22], and have distinct but overlapping functions, as inferred from their mutant phenotypes [8,9]. Whereas wild type *A. majus *plants have five petals, four stamens and a dorsal staminode, *cyc *single mutants often have extra dorsal petals that are reduced in size, and a fully developed dorsal stamen. By contrast, *dich *single mutants only lack the internal asymmetry of wild type dorsal petals. Together with the fully ventralized, and, hence, radially symmetrical flowers of *cyc:dich *double mutants, these data demonstrate a role for *CYC *in dorsal stamen abortion, petal growth and organ number determination, and a role for *DICH *in shaping dorsal petal growth [8,9].

A similar range of dorsal identity functions has been assigned to *CYC*-like genes of other core eudicots, including *Linaria vulgaris, P. sativum, I. amara, Lotus japonicus*, and *G. hybrida *[10-13,20,23]. Furthermore, although monocots do not have a strict *CYC *gene ortholog due to two separate duplication events at the base of core eudicots [24], the observation that the class II TCP gene *RETARDED PALEA1 (REP1) *in *Oryza sativa *(rice, Poaceae) is expressed only in the dorsally positioned palea, suggests further recruitment of TCP genes in the evolution of monocot flower bilateral symmetry [17]. Indeed, it was recently found that bilaterally symmetrical flowers of *Costus spicatus *(Costaceae; Zingiberales) and *Heliconia stricta *(Heliconiaceae) have asymmetric TCP gene (*CsTB1a *and *HsTBL2b*, respectively) expression at early to late stages of flower development [18]. However, in contrast to the core eudicots, *CsTB1a *and *HsTBL2b *expression is restricted to the ventral, rather than the dorsolateral, side of the flower [18].

In addition to a hypothesized role for TCP genes, the floral homeotic B-class genes *DEFICIENS *(*DEF*) and *GLOBOSA *(*GLO*) have been implicated in the multiple independent derivations of flower bilateral symmetry in monocots [25-29]. Evidence supporting this comes largely from the observation that *DEF*-like gene paralogs are differentially expressed in the morphologically distinct outer tepals, dorsolateral inner tepals and ventral inner tepal (lip or labellum) of *Phalaenopsis equestris *(Orchidaceae) [25,26]. In both core eudicots and monocots, DEF- and GLO-like proteins function as obligate heterodimers to confer identity to the second and third whorls, although there are exceptions [30,31]. In the second whorl of *Arabidopsis thaliana *(Brassicaceae) and *A. majus *APETALA3 (AP3)/DEF and PISTILLATA (PI)/GLO form tetramers with SEPALLATA (SEP) and APETALA1 (AP1)/SQUAMOSA (SQUA) MADS-box proteins, resulting in the activation of downstream genes that confer petal identity [32-36]. Thus, since *DEF/GLO*-, and to a lesser extent *SEP*- and *SQUA*-like, gene function is largely conserved across angiosperms [37-45], shifts between radial and bilateral perianth symmetry could be explained by changes in expression of these floral homeotic genes, resulting in the loss, gain or modification of second whorl identity.

**Figure 1:**
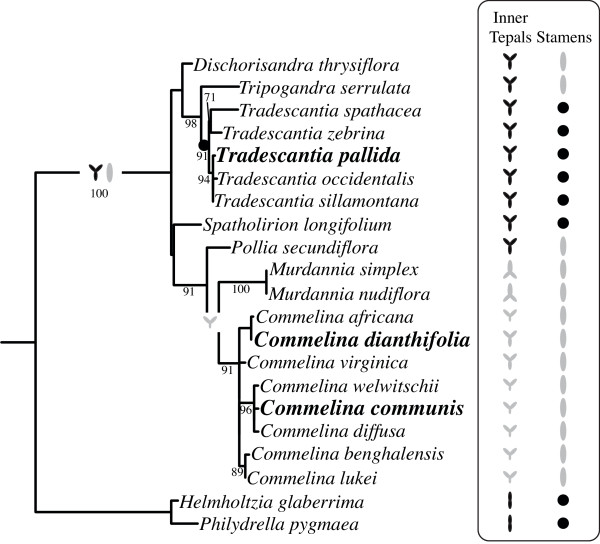
**Evolution of inner tepal and stamen symmetry in Commelinaceae**. Maximum likelihood (ML) *trn*L phylogeny reconstruction with support values for 500 ML bootstrap replicates based on Burns *et al*. (2011), using Philydraceae species as outgroups [55]. Focal taxa are in bold. Inner tepal and stamen morphology is depicted for each species as bilaterally symmetrical (gray) or radially symmetrical (black). The ancestral state of Commelinaceae inner tepal and stamen symmetry is reconstructed as radial and bilateral, respectively. A shift to radial stamen symmetry is inferred at the base of *Tradescantia*, whereas a late-developmental shift to weak bilateral symmetry is inferred for *C. dianthifolia *and *C. virginica*.

The monocot order Commelinales contains species with both radially and bilaterally symmetrical flowers, is sister to the Zingiberales and comprises five families: Commelinaceae (for example, *Commelina *and *Tradescantia*), Hanguanaceae (*Hanguana *only), Philydraceae (for example, *Philydrella *and *Helmholtzia*), Haemodoraceae, and Pontederiaceae [46,47] (Figure 1). Phylogenetic analyses suggest that showy, insect-pollinated and bilaterally symmetrical flowers are plesiomorphic to the Commelinales/Zingiberales clade [47]. However, since *Hanguana *and *Cartonema *(Commelinaceae) are both progressively sister to the two major Commelinaceae clades, and have radially symmetrical flowers, it has been suggested that radial flower symmetry is ancestral to Commelinaceae [29,48]. Based on the prevalence of radial flower symmetry in its sister families - including Joinvilleaceae and Flagellariaceae - an ancestral state of bilateral flower symmetry has similarly been invoked for Poaceae [48-50]. Thus, flower bilateral symmetry probably evolved independently in the Commelinaceae (Figure 1), Philydraceae, Zingiberales and Poaceae, as well as in other monocots outside the commelinid clade (for example, Orchidaceae) [29]. A critical question in evolutionary developmental biology is whether similar changes at the level of gene networks, genes and/or gene regions underlie these convergent shifts in floral form.

**Figure 2:**
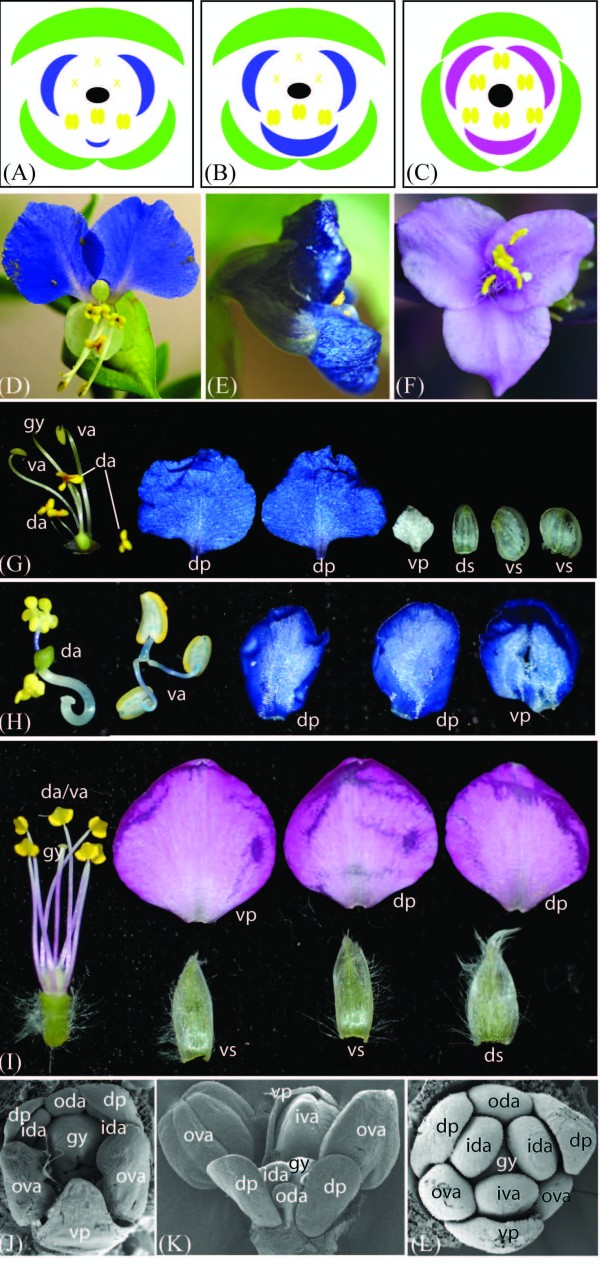
**Flower morphological diversity in representative Commelinaceae species**. **(A-C) **Floral diagrams of mature *C. communis *(A), *C. dianthifolia *(B) and *T. pallida *(C) flowers. Stamens (yellow) and gynoecia (black) of both *Commelina *species and inner tepals (blue or pink) of *C. communis *are strongly bilaterally symmetrical, outer tepals (green) of both *Commelina *species and inner tepals of *C. dianthifolia *are weakly bilaterally symmetry, and all organ whorls of *T. pallida *are nearly radially symmetry. **(D) **Front view of a bilaterally symmetrical *Commelina communis *flower showing the two large dorsal inner tepals (blue), reduced ventral inner tepal, reduced dorsal staminodes (upper yellow organs), and pollen-producing ventral stamens (lower yellow organs). **(E) **Side view of a *C. dianthifolia *flower showing the two large dorsal inner tepals (upper blue) and the even larger ventral inner tepal (lower blue). **(F) **Front view of a radially symmetrical *Tradescantia pallida *flower showing the three equal pink inner tepals, and six equal stamens. **(G) **Individual organs of post-anthesis *C. communis *flowers. **(H) **Individual organs of post-anthesis *C. dianthifolia *flowers; outer tepals are not shown. **(I) **Individual organs of post-anthesis *T. pallida *flowers. **(J) **Scanning electron micrograph (SEM) of a mid-stage *C. communis *flower; the ventral region develops faster than the dorsal region (this stage), and then the ventral inner tepal arrests development (later stage). **(K) **SEM of a late stage *C. dianthifolia *flower. The ventral region develops faster than the dorsal region from early to late stages. **(L) **SEM of an early stage *T. pallida *flower showing that radial symmetry is established early in development. ds, dorsal outer tepal; ventral outer tepal; dp, dorsal inner tepal; vp, ventral inner tepal; oda, outer dorsal androecium; ida, inner dorsal androecium; iva, inner ventral androecium; ova, outer ventral androecium; da; dorsal androecia; va, ventral androecia; gy, gynoecium.

Flower bilateral symmetry in the genus *Commelina *involves differential organ development in all of the whorls (Figures 1 and 2) [51-55]. In *C. communis *and *C. dianthifolia*, each hermaphroditic flower comprises two whorls of three tepals, two whorls of three stamens/staminodes and a whorl of three fused carpels (Figure 2A, B) [54]. In the first whorl of both species, all three outer tepals are membranous, but the lateroventral two are distinct from the dorsal one in both size and shape (Figure 2G). In the second whorl of *C. dianthifolia *all inner tepals are large and showy, but vary slightly in overall size and shape along the dorsoventral axis (Figure 2E, H). By contrast, in the second whorl of *C. communis*, only the dorsolateral inner tepals are large and showy; the ventral inner tepal is small and membranous, similar to outer tepals (Figure 2D, G) [54]. In the stamen whorls of both species, the dorsal (outer whorl) or dorsolateral (inner whorl) organs are underdeveloped staminodes that function to attract pollinators, but produce few sterile pollen grains (Figure 2D, G, H). By contrast, the long lateroventral stamens in the outer whorl, and the medium ventral stamen in the inner whorl are showy and produce viable pollen (Figure 2D, G, H). Finally, in the fifth whorl of both species, the dorsal carpel is underdeveloped and sterile, while the two ventral carpels are fertile [54].

Based on detailed analyses of flower development, and the known role of class II TCP genes in core eudicots, Hardy *et al*. (2009) recently hypothesized a role for *TB1*-like TCP genes in establishing bilateral symmetry in early developmental stages of *Commelina *flower development [54]. Furthermore, we propose a second, non-exclusive hypothesis, that dorsal-specific expression of *DEF/GLO*-like genes defines the dorsoventral axis of symmetry in mid- to late-stage *C. communis *corollas. In this study, we use gene expression and micromorphological data to test three predictions of these hypotheses that: (1) *TB1-*like genes are asymmetrically expressed in bilaterally symmetrical *C. communis *and *C. dianthifolia*, but not radially symmetrical *Tradescantia *(formerly *Setcreasea*) *pallida*, corollas consistent with a role in differential organ growth, (2) *DEF *and *GLO *orthologs are co-expressed in staminodes, stamens and the dorsolateral inner tepals, but not in outer tepals or the outer tepal-like ventral inner tepal of *C. communis*, and (3) cellular morphology of the *C. communis *ventral inner tepal is more similar to outer tepals than dorsolateral inner tepals, suggesting asymmetric loss of inner tepal identity in the second whorl.

## Methods

### Plant material

Inflorescence material was harvested from wild-collected, flowering individuals of *C. communis *and *T. pallida *in Missouri and Kansas (USA) during the summer months, and from *C. dianthifolia *plants grown under standard greenhouse conditions at the University of Kansas. For scanning electron microscopy (SEM) and *in situ *hybridization, whole inflorescences were fixed overnight in formalin acetic alcohol (FAA: 47.5% (v/v) ethanol, 5% (v/v) acetic acid, 3.7% (v/v) formaldehyde), and gradually taken through an alcohol series to 100% ethanol. For quantitative reverse transcriptase polymerase chain reaction (qRT-PCR), flower organs from 10 to 16 flowers were individually dissected twice from 3 to 5 mm long buds, pooled according to identity/position, and snap-frozen in liquid nitrogen. Total RNA was extracted using TriReagent (Applied BioSystems, Carlsbad, CA, USA), DNA contamination was removed using TURBO DNase (Applied BioSystems), and 1 μg of RNA was reverse transcribed using the iScript Select cDNA synthesis kit (Bio-Rad, Hercules, CA, USA). Genomic DNA was extracted from leaf material using the DNeasy Plant Mini Kit (Qiagen, Valencia, CA, USA).

### SEM

Ethanol-dehydrated inflorescences were dissected to reveal internal floral organs as necessary. Tissues were critical point dried in a Tousimis critical point drier, mounted on stubs, sputter-coated with gold and viewed with a D. Leo field emission scanning electron microscope (VTT, Espoo, Finland). For micromorphological analyses of inner and outer tepals, both adaxial and abaxial surfaces were analyzed at regions proximal, medial and distal to the floral axis.

### Gene isolation and phylogenetic analysis

In order to isolate and sequence all *TB1*-like orthologs from genomic DNA of *C. communis*, *C. dianthifolia *and *T. pallida*, we used the degenerate forward primers, CYCF1, CYCF2, CYC73aaF, CYC73bF, which were previously designed to amplify *CYC/TB1 *genes from a wide range of angiosperm taxa, in combination with the reverse primers CYCR1 and CYCR2 [56,57]. Amplicons were cloned into the pGEM-T vector (Bio-Rad), and 5 to 10 colonies per successful PCR reaction were sequenced. To determine if the Commelinaceae sequences corresponded to *CYC/REP/TB1*-like genes, and to determine orthology and copy number, amino acid sequences spanning the TCP to R domain from *C. communis*, *C. dianthifolia *and *T. pallida *were aligned by MAAFT [58,59] and then by eye in MacClade [60] with previously designated *CINCINATTA *(*CIN*)-like, *CYC*-like, *REP*-like and *TB1*-like TCP genes [24,61]. Nucleotide alignments were subjected to maximum likelihood (ML) analyses in GARLI 0.951 and Bayesian analyses in MrBayes 3.1.2 following model optimization in MrModelTest [62-64]. ML analyses were run using 10 random addition sequences with 500 bootstrap replicates. Bayesian analyses were run twice for 10 million generations, sampling every 1,000 generations, with 25% of trees discarded as burn-in. Newly generated *TB1*-like sequences were submitted to Genbank with accession numbers [Genbank: JQ622131-JQ622135 and JQ622142].

*DEF *and *GLO *orthologs were isolated and sequenced from cDNA of *C. communis*, *C. dianthifolia *and *T. pallida *using previously described degenerate primers [65]. Amplicons were cloned and sequenced as previously described, and aligned nucleotide sequences were subjected to ML and Bayesian analyses as described for the *TB1*-like genes. Newly generated *DEF*- and *GLO*-like genes were submitted to Genbank with accession numbers [Genbank: JQ622136-JQ622141].

### qRT-PCR

To compare patterns of *TB1*-like and B-class gene expression in mid-stage floral organs of *T. pallida*, *C. dianthifolia *and *C. communis*, qRT-PCR analyses using DyNAzyme II Hot Start DNA Polymerase (GE Healthcare, Pittsburgh, PA, USA) and SYBR Green I (Invitrogen, Carlsbad, CA, USA) were conducted on a DNA Engine Opticon 2 real-time PCR machine (MJ Research. Waltham, MA, USA) as previously described [66]. Two primers pairs per target gene were designed in Primer 3, and the most efficient primer pair was selected for expression analysis (Additional file 1) [67]. Where possible, primer pairs were designed to span introns to rule out DNA contamination. β-*ACTIN *(*ACT*) and *EUKARYOTIC TRANSLATION ELONGATION FACTOR 1 ALPHA 1 *(*EF1alpha*) showed little transcriptional variation across different tissues of *C. communis *and *C. dianthifolia*, and *T. pallida*, respectively, and were, therefore, selected as reference genes. A negative cDNA control containing RNA from dorsolateral inner tepals and lacking the reverse transcriptase enzyme was initially used as a negative control template for all qRT-PCR analyses. Furthermore, when no transcription was detected in floral material, genomic DNA was used as a positive control. For each gene the mean and standard deviation was determined for three to four technical replicates. Results were analyzed separately for one (*C. dianthifolia*) or two (*C. communis *and *T. pallida*) biological replicates to account for differences in developmental staging of the pooled material, and compared for consistent results between organ type and organ position. Analyses were not carried out for *C. dianthifolia *outer tepals due to difficulty obtaining good quality RNA from these organs.

### *In situ *hybridization

To better determine the spatio-temporal pattern of gene expression, *in situ *hybridization was carried out in wax-embedded inflorescence tissues of the most closely related species, *C. communis *(*DEF*, *GLO *and *TB1a*) and *C. dianthifolia *(*DEF *and *TB1a*). Antisense and sense gene-specific probes of *CcDEF *(for both species)*, CcGLO *(*C. communis *only)*, CcTB1a *(*C. communis *only) and *CdTB1a *(*C. dianthifolia *only) were generated using T7 and SP6 RNA Taq polymerase (Roche Applied Science, Indianapolis, IN, USA) according to the manufacturer's instructions. Each probe spanned ca. 400 bps, and included part of the C-terminal domain and the 3'-untranscribed region. As an experimental control, an antisense probe was generated for *CcHistone4 *as described previously [57]. *In situ *hybridization was performed as described previously [68,69].

## Results

### Flower development and perianth micromorphology

Comparative morphological analyses revealed a similar progression of flower development for *C. communis *and *C. dianthifolia *at early to mid stages. In both species, development proceeded asymmetrically, with the ventral/lateroventral organs developing more rapidly than the dorsal/dorsolateral organs (Figure 2J, K). In the outer tepal and stamen whorls, this asymmetry was maintained into late stage development for both species (Figure 2D, E, G, J, K). By contrast, at mid to late stages of development the ventral inner tepal of *C. communis*, but not *C. dianthifolia*, arrested prematurely. This resulted in strong asymmetry in the mature corolla of *C. communis*, with the ventral inner tepal resembling the small colorless outer tepals (Figure 2D, G versus 2E, H). Unlike *C. communis *and *C. dianthifolia*, flowers of *T. pallida *were radially symmetrical from early to late stages of development (Figure 2F, I, L).

**Figure 3:**
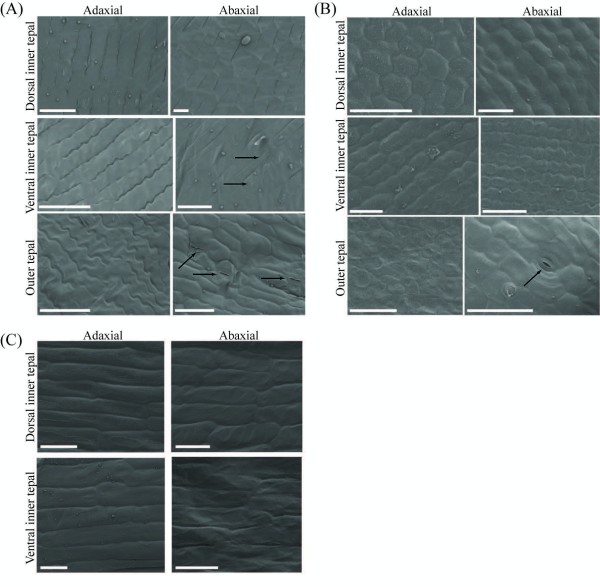
**Cellular micromorphology of *Commelina *tepals**. **(A) **The adaxial surfaces of *Commelina communis *inner and outer tepals are characterized by long cells with wavy margins; whilst the abaxial surfaces have cells with straighter edges. The abaxial surfaces of the ventral inner tepal and the outer tepals are similar regarding the presence of stomata, which are absent from the abaxial surface of dorsal inner tepals. **(B) **Stomata are only found on the abaxial surface of outer tepals from *Commelina dianthifolia*. **(C) **Stomata are lacking from the abaxial and adaxial surfaces of both dorsal and ventral inner tepals of *Tradescantia pallida*. Scale bars denote 50 μm. Arrows indicate closed (*C. communis *ventral inner tepals), partially closed (*C. communis *outer tepals), and open stomata (*C. dianthifolia *outer tepals).

To determine if the reduced outer tepal-like ventral inner tepal of *C. communis *also resembled outer tepals at the micromorphological level, detailed SEM analyses were conducted on mature floral organs at the cellular level. *C. communis *outer tepals, dorsolateral inner tepals and ventral inner tepals were all characterized by long wavy cells on both the adaxial and abaxial surfaces (Figure 3A). Additionally, the abaxial surface of both outer tepals and ventral inner tepals had stomata; no stomata were found on either surface of the dorsolateral inner tepals, or the adaxial surface of outer tepals and ventral inner tepals (Figure 3A). To distinguish between the alternative possibilities that stomata define outer tepal identity or determine ventral inner tepal identity, similar analyses were conducted on mature *C. dianthifolia *(both outer and inner tepals) and *T. pallida *(only inner tepals) flowers (Figure 3B, C). As in *C. communis*, *C. dianthifolia *outer tepals were marked by stomata on the abaxial surface. However, unlike *C. communis *ventral inner tepals, no stomata were evident on either surface of the dorsolateral and ventral inner tepals in *C. dianthifolia *or *T. pallida *(Figure 3B, C).

### Gene duplication in the *TB1*-like gene lineage

**Figure 4:**
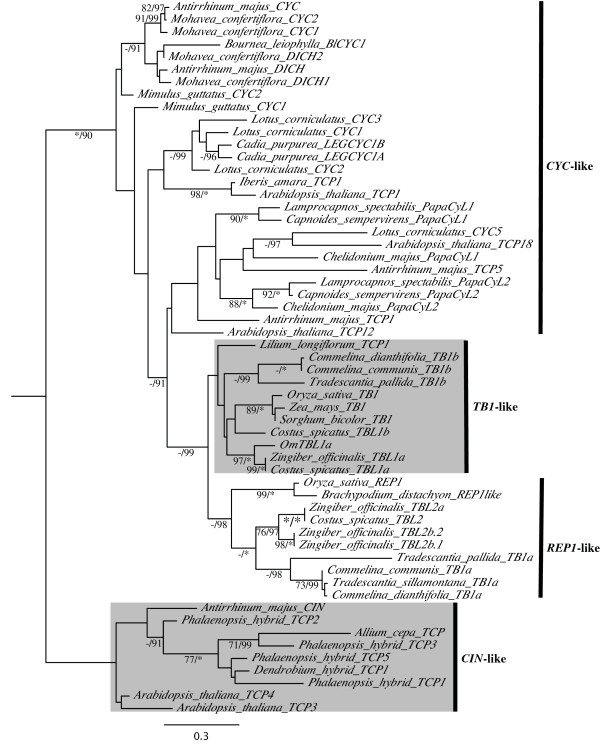
**Best maximum likelihood phylogeny of monocot *TB1*-like genes rooted with core eudicot *CIN-*like genes**. Maximum likelihood bootstrap values above 69% (left) and Bayesian posterior probabilities above 89% (right) are shown for each branch. Asterisks denote 100% support. Dashes indicate bootstrap support below 70% only when the corresponding posterior probability is shown. The *TB1*-like and *CIN*-like gene clades are shaded.

Cloning and phylogenetic analyses revealed two *CYC/REP/TB1*-like homologs (hereafter *TB1a *and *TB1b*) in *C. communis*, *C. dianthifolia *and *T. pallida *that formed two distinct clades (Figure 4). Maximum likelihood and Bayesian analyses yielded single trees with similar topologies that largely tracked the known species phylogeny of monocots. The Commelinaceae *TB1a *genes formed a well-supported clade (73% ML bootstrap (MB); 99% posterior probability (PP)) sister to the *TBL2 *genes of Zingiberales species (100% PP), and together these clades were sister to the *REP1 *genes in Poaceae (98% PP) (Figure 4). Thus, orthology between Commelinacae *TB1a*, Zingiberales *TBL2 *and Poaceae *REP1 *genes was strongly supported by Bayesian PPs. Commelinaceae *TB1b *genes similarly formed a well-supported clade (99% PP) sister to the *TB1*-like genes of Zingiberales, Poaceae and Liliaceae species. However, the relationship between genes of each family was not well-supported (Figure 4). On a broader scale, both *REP1 *and *TB1 *clades grouped with *CYC*-like genes from eudicots (100% MB; 90% PP) (Figure 4), even when the more distantly related *PCF*-like TCP genes were used as outgroups [61] (data not shown).

### Differential expression of *TB1*-like genes

**Figure 5:**
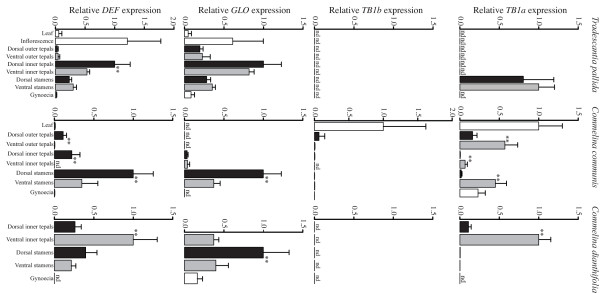
**Relative quantitative gene expression in dissected flower tissues**. *TB1a *expression is significantly stronger in ventral versus dorsal tepals of *C. communis *and *C. dianthifolia *but not *T. pallida*, and is expressed asymmetrically in different directions in stamens of all three species (top panel). *TB1b *expression is low or absent in floral tissues, but is expressed strongly in leaves (second top panel). *GLO *expression is significantly stronger in staminodes versus stamens of *C. dianthifolia *and *C. communis*, and is low to undetectable in outer tepals and gynoecia (second bottom panel). *DEF *expression is significantly stronger in dorsal versus ventral inner tepals of *C. communis*, being undetectable in the latter (bottom panel). *β-ACT *and *EF1a *were used as internal controls. Experiments were conducted with three to four technical replicates. Bars show mean and standard deviation. Second biological replicates were analyzed separately and are shown in Additional file 2. White bars, whole organs; blacks bars, dorsal organs; gray bars, ventral organs. Asterisks denote significant differences between dorsal and ventral organ comparisons. nd, not detected.

In order to test the prediction that *TB1 *orthologs are expressed asymmetrically in mid stage bilaterally symmetrical *C. communis *and *C. dianthifolia *tepals, but not radially symmetrical *T. pallida *tepals, expression analyses were carried out on dissected flower buds using qRT-PCR (Figure 5 and Additional file 2). In *C. communis*, *TB1a *expression was significantly higher in ventral versus dorsolateral outer tepals (average 10.4-fold difference (SD = 9.7) for two biological replicates), and ventral versus dorsolateral inner tepals (average 12.6-fold difference (SD = 9.7) for two biological replicates) (Figure 5 and Additional file 2). Similarly, in *C. dianthifolia*, *TB1a *expression was significantly higher in ventral versus dorsolateral inner tepals (9.1-fold difference for one biological replicate) (Figure 5). No *TB1a *expression was detected in either outer or inner tepals of *T. pallida *for either biological replicate (Figure 5 and Additional file 2).

Unlike tepals, the correlation between *TB1a *expression and stamen differentiation was only weakly supported. In *C. communis*, although *TB1a *was asymmetrically expressed in dorsal staminodes versus ventral stamens, the direction of differential expression contrasted between replicates (Figure 5 and Additional file 2). This suggests dynamic expression of *TB1a *at mid stage stamen development. By contrast, *TB1a *expression was weak but consistently higher in dorsal staminodes versus ventral stamens of *C. dianthifolia *(2.3-fold difference for biological replicate one (Figure 5); detectable in dorsal staminodes but undetectable in ventral stamens for biological replicate two (data not shown)), and was not significantly different between dorsal and ventral stamens of *T. pallida *(1.2-fold difference for one biological replicate) (Figure 5). In contrast to qRT-PCR analyses, no *TB1a *expression was detectable in early to late stage flowers of *C. communis*, *C. dianthifolia *or *T. pallida *using *in situ *hybridization (data not shown). This suggests that *TB1a *transcripts are below the level of detection using this method.

QRT-PCR analyses of *TB1b *genes revealed different expression patterns relative to *TB1a *paralogs (Figure 5 and Additional file 2). In *T. pallida*, *TB1b *was undetectable in all floral organs and leaves, despite amplification of genomic DNA (Figure 5 and Additional file 2). In *C. communis*, *TB1b *expression was highest in leaves, with low (Figure 5) to no (Additional file 2) detectable expression in all other organs. For both replicates, no significant differences in transcript levels were detected between dorsal and ventral organs (Figure 5 and Additional file 2). Finally, for *C. dianthifolia*, no *TB1b *expression was detected in any floral organ, again despite amplification of genomic DNA (Figure 5).

### Differential expression of B-class genes

To test the prediction that B-class gene expression is exclusively reduced or absent from the outer tepal-like ventral inner tepal of *C. communis*, *DEF *and *GLO *genes were isolated from all three focal species, and both qRT-PCR and *in situ *hybridization analyses were carried out on inflorescence tissues. ML and Bayesian analyses supported the isolation of both *DEF *and *GLO *orthologs from each species, and relationships among a larger set of *DEF *and *GLO *genes largely tracked the known species tree for monocots (Additional file 3). As predicted, *GLO *was expressed in inner tepals and stamens of *C. communis, C. dianthifolia *and *T. pallida *(Figure 5 and Additional file 2). Furthermore, analyses revealed low (*T. pallida *based on one biological replicate) to no (*C. communis *based on two biological replicates) expression in outer tepals of representative *Tradescantia *and *Commelina *species (Figure 5 and Additional file 2). In *C. communis*, *GLO *was expressed similarly in dorsolateral and ventral inner tepals, with expression being significantly higher in dorsal staminodes than ventral stamens (2.1-fold difference for two biological replicates (SD = 0.27)) (Figure 5 and Additional file 2). A similar asymmetric expression pattern was detected in the stamen whorls of *C. dianthifolia *(Figure 5). However, in *T. pallida *there was no significant difference in expression between ventral versus dorsal inner tepals and stamens for one biological replicate (Figure 5 and Additional file 2).

**Figure 6:**
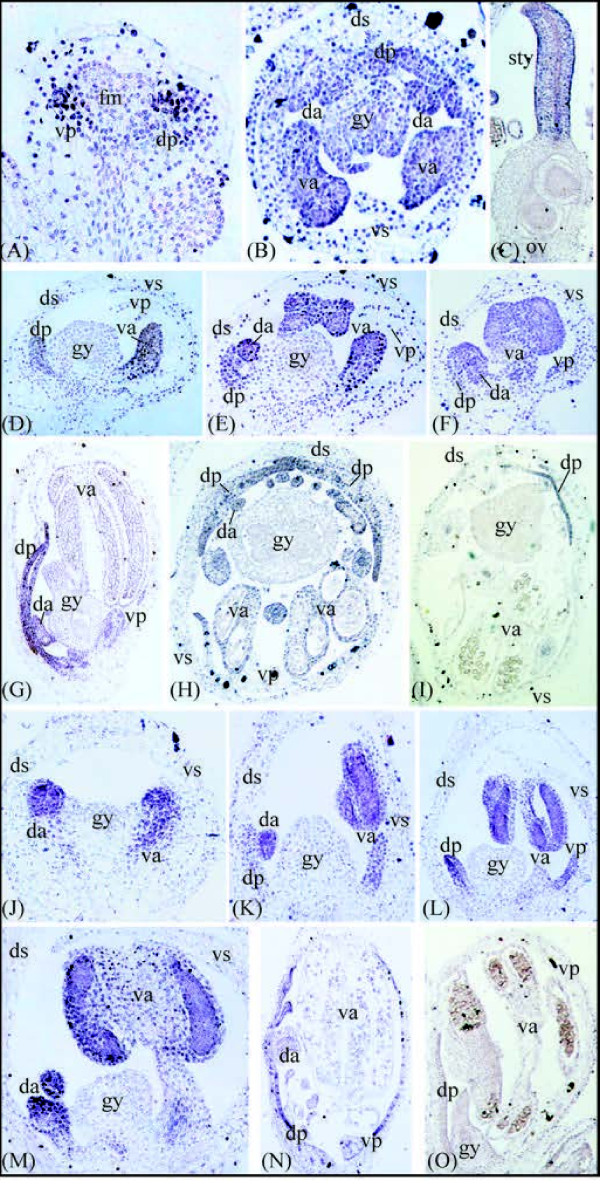
***In situ *hybridization of B-class genes in *Commelina communis *and *C. dianthifolia***. **(A-C) ***C. communis *flower sections showing expression of *GLOBOSA *(*GLO*) in early to late stages of inner tepal and stamen/staminode development, and late stages of gynoecial development. **(D-F) **Mid-stage *C. communis *flowers sectioned longitudinally showing *DEFICIENS *(*DEF*) expression in the dorsal inner tepals, dorsal staminodes, and ventral stamens. No transcripts were detected in the outer tepals, ventral inner tepals, or gynoecium. **(G) **Late-stage longitudinal section of a *C. communis *flower showing *DEF *expression in the dorsal inner tepals and staminodes. **(H-I) **Transverse sections through late-stage *C. communis *flowers showing *DEF *expression in the dorsal inner tepals, dorsal staminodes, and ventral stamens. **(J) **Early-stage *C. dianthifolia *flower showing comparable *DEF *expression in the dorsal and ventral regions of the stamen primordia. **(K-M) **Mid-stage *C. dianthifolia *flowers showing *DEF *expression in the dorsal inner tepals, dorsal staminodes, ventral inner tepals, and ventral staminodes. **(N) **Late-stage *C. dianthifolia *flower showing *DEF *expression in all inner tepals and the dorsal staminodes. **(O) **Sense *DEF *control in *C. communis *shows little to no staining. fm, floral meristem; ds, dorsal outer tepal; vs, ventral stamen; dp, dorsal inner tepal; vp, ventral inner tepal; da, dorsal androecium; va, ventral androecium; gy, gynoecium; sty, style; ov, ovary.

Consistent with qRT-PCR analyses, *in situ *hybridization analyses revealed *GLO *expression in the inner tepal and stamen whorls of young *C. communis *flower buds, with transcripts being absent from the center of the flower (Figure 6A). The second to fourth whorl expression was maintained during mid to late stages of *C. communis *flower development, with transcripts becoming detectable in the gynoecium from mid to late stages (Figure 6B, C). Since qRT-PCR for all three species, and *in situ *hybridization for *C. communis*, revealed no correlation between *GLO *expression and inner tepal morphology, *in situ *hybridization experiments were not carried out for *C. dianthifolia *or *T. pallida GLO *orthologs.

QRT-PCR analyses revealed *DEF *gene transcripts in inner tepals and stamens of *C. communis, C. dianthifolia *and *T. pallida *(Figure 5 and Additional file 2), with low to no expression of *DEF *in outer tepals and gynoecia of *C. communis *and *T. pallida *(Figure 5 and Additional file 2). In *C. communis*, *DEF *expression was significantly higher in dorsal staminodes versus ventral stamens (3.5-fold difference for two biological replicates (SD = 0.32)), and in contrast to dorsolateral inner tepals, was completely absent from ventral inner tepals for two biological replicates (Figure 5 and Additional file 2). In *C. dianthifolia, DEF *expression was not significantly different between dorsal staminodes and ventral stamens. However, in striking contrast to *C. communis*, *DEF *was significantly higher in ventral versus dorsolateral inner tepals (2.3-fold difference for one biological replicate). Finally, in *T. pallida*, *DEF *expression was not significantly different between dorsal and ventral stamens, and was highly variable in dorsolateral versus ventral inner tepals between two biological replicates (Figure 5 and Additional file 2).

Consistent with qRT-PCR, *in situ *hybridization analyses of *DEF *showed much higher expression levels in dorsal compared to ventral inner tepals of early to late stage *C. communis *flower buds (Figure 6D-I). However, in the stamen whorls, this dorsoventral gradient of *DEF *expression only became evident following late-stage differentiation of the ventral stamens (Figure 6D-I). Comparable analyses in *C. dianthifolia *revealed *DEF *expression in both dorsolateral and ventral inner tepals, dorsal staminodes and ventral stamens during early to midstages of development (Figure 6J-M). Strong expression was maintained in both the dorsolateral and ventral inner tepals into late development (Figure 5N). However, *DEF *expression became gradually weaker in ventral stamens relative to dorsal staminodes during late stage anther differentiation (Figure 6M, N). No antisense transcripts of *DEF *were detectable in outer tepals or gynoecia of *C. communis *or *C. dianthifolia*. Furthermore, no staining was observed in control sections of either species using *DEF *or *GLO *sense probes (Figure 6O and data not shown).

## Discussion

A major conclusion from this study is that *TB1*-like genes have been independently recruited in establishing Commelinaceae corolla bilateral symmetry in early development, similar to several core eudicot lineages and rice [8-13,17]. Furthermore, micromorphological evidence and expression of the B-class MADS-box gene *CcDEF *suggests that late stage asymmetry between the dorsolateral and ventral inner tepals of *C. communis *is due to a homeotic transformation of the ventral inner tepal into an outer tepal. In the sections below, we discuss the implications of these results as they relate to our understanding of convergent trait evolution.

### Parallel evolution of *TB1*-like genes across angiosperms

Bilateral flower symmetry can be achieved through the asymmetric loss or reduction of organs (structural zygomorphy) or the modification of organs (presentation zygomorphy) within one or more whorls, and can be present in early and/or late stages of development [48]. Although both types of zygomorphy are found within the monocots, structural zygomorphy is particularly prevalent within the Asparagales (for example, Orchidaceae), Arecaceae, Dasypogonaceae, Zingiberales (for example, Zingiberaceae), Commelinales (for example, Commelinaceae) and Poales (for example, Poaceae) [18,27,29,48,70]. In most cases, bilateral symmetry affects the inner tepals and stamen whorls. However, outer tepal, inner tepal, stamen and gynoecial zygomorphy have all evolved in the monocots multiple times independently [48].

Maximum parsimony character state reconstructions support a gain of tepal bilateral symmetry within the *Commelina*-containing Commelineae clade of Commelinaceae (Figure 1). Available data suggest that members of the Commelineae have flowers that are strongly bilaterally symmetrical in early development, but vary in the strength of structural tepal zygomorphy in late development [54,71] (this study). For example, although all are strongly zygomorphic in early development, at anthesis *C. communis *inner tepals are highly differentiated along the dorsoventral axis, whereas *C. dianthifolia *and *Murdannia nudiflora *inner tepals are only slightly differentiated in size and shape. Based on evidence from core eudicots, it has been hypothesized that asymmetric expression of *CYC/TB1*-like genes underlies early stage structural zygomorphy in the Commelineae [54]. Consistent with this hypothesis, *TB1a *is expressed asymmetrically in tepals of *C. communis *and *C. dianthifolia*, but not *T. pallida*, at mid stages of flower development. However, in contrast to the asymmetric expression of *CYC *and *DICH *in the model core eudicot *A. majus *and *REP1 *in *O. sativa*, expression is significantly higher in the ventral, as opposed to dorsolateral, tepals [8,9,17] (this study). It is predicted that the asymmetrical expression of *TB1a *in *C. communis *and *C. dianthifolia *is initiated in early flower development and has no effect on late stage development when the ventral inner tepal of *C. communis *arrests growth (see next section).

In *A. majus *expression of *CYC *in the dorsal floral meristem represses growth; a similar repressive function has been assigned to *IaCYC *of the rosid core eudicot *Iberis amara *(Brassicaceae) in mid to late stage dorsal petal development [8,9,11]. By contrast, at mid to late stages of *A. majus *flower development, *CYC *expression in the dorsal petals actually increases cell proliferation and elongation relative to the ventral petal [8,9]. Together with results from Commelinaceae, these data support developmentally and taxonomically distinct roles for *CYC *in establishing early to late stage perianth organ growth, and suggest that parallel recruitment of *CYC/TB1 *genes in floral bilateral symmetry is not limited to the dorsal side of the flower. Indeed, a ventral pattern of expression was recently demonstrated for *CsTBL1a *in bilaterally symmetrical *C. spicatus *(Costaceae, monocot) flowers [18].

Further studies testing the involvement of *CYC/TB1 *genes in transitions from radial to bilateral flower symmetry will require functional tests, and should aim to elucidate whether changes in *CYC/TB1 *expression are due to *cis*-regulatory or upstream changes. In *A. majus*, the NAC family protein *CUPULIFORMIS (CUP*) has been implicated in the positive regulation of *CYC *[72]. Thus, expression analyses of *CUP*-like genes in perianth organs of Commelinaceae and other monocots varying in flower symmetry might be a good starting point to address this question.

### Parallel evolution of B-class genes in monocots

It has been hypothesized that changes in the expression of organ identity genes can explain the evolution of flower bilateral symmetry in certain monocots by altering the identity of a subset of organs within a floral whorl. In the case of orchids, one of several *DEF*-like genes evolved an asymmetric expression pattern following gene duplication, and is implicated in modification of the ventrally positioned inner tepal into the characteristic lip [25,27,73-75]. Our data also support a role for *DEF*-like gene evolution in modification of the *C. communis *ventral inner tepal. However, unlike orchids, the modification of *DEF*-like gene expression in *C. communis *was not preceded by gene duplication and is presumably not associated with changes in protein function at the biochemical level. Furthermore, unlike *C. dianthifolia *and *T. pallida*, the ventral inner tepals of *C. communis *flowers have stomata on their abaxial surface similar to outer tepals. This micromorphological marker correlates both with the general outer tepal-like appearance of the ventral inner tepal and the complete absence of *DEF*-like gene expression during early to late stages of development.

Together, these studies suggest parallel evolution of *DEF*-like genes in the independent origins of monocot flower bilateral symmetry. Future studies in other monocot families will be required to determine the generality of these results, and to test whether evolution of *DEF*-like gene expression can explain petaloidy in the stamen whorl [29]. Furthermore, a key question remains as to whether evolution of *DEF*-like gene expression can be explained by *cis*-regulatory or upstream changes. In *A. thaliana*, SUPERMAN (SUP), FLORAL ORGAN NUMBER1 (FON1), CUP-SHAPED COTYLEDON1 (CUC1), and CUC2 proteins negatively regulate both B-class genes in the sepal whorl [76]. Thus, expression of these proteins in the ventral inner tepal of *C. communis *could explain the loss of *DEF*-like expression in this organ. However, this scenario would require that SUP-, FON1- and CUC1/2-like proteins do not regulate the *C. communis GLO*-like gene, which is expressed normally in the ventral inner tepal.

## Conclusions

Together with other studies, our gene expression data on three morphologically diverse species of Commelinaceae suggest a parallel role for two major transcription factors in the independent evolution of angiosperm flower bilateral symmetry. In the case of class II TCP genes, changes in expression are correlated with early developmental shifts from radial to bilateral flower symmetry in several core eudicots and commelinid monocots (rice, *Costus *and *Commelina*). This supports a conserved role for class II TCP genes in organ growth across angiosperms, and suggests either evolutionary constraint on the flower symmetry gene network or the involvement of few genes in the establishment of floral meristem symmetry. Evolution of *DEF*-like genes is also implicated in shifts from radial to bilateral flower symmetry, at least within divergent monocots (*Phaelaenopsis *and *C. communis*). However, in contrast to class II TCP genes, changes in both function (*Phaelaenopsis*) and regulation (*C. communis*) of B-class genes are implicated in late developmental shifts in within-whorl organ identity. Further studies are required to test the generality of class II TCP and B-class gene evolution in diversification of monocot flowers, and to decipher whether/why the same genes have been the targets of repeated selection across millions of years of angiosperm diversification.

## Abbreviations

*ACT: β-ACTIN; DEF: DEFICIENS; CIN: CINCINATTA; CUC1: CUP-SHAPED COTYLEDON1; CUP: CUPULIFORMIS; CYC: CYCLOIDEA; DICH: DICHOTOMA; EF1alpha: EUKARYOTIC TRANSLATION ELONGATION FACTOR 1 ALPHA 1; *FAA: formalin acetic alcohol; *FON1: FLORAL ORGAN NUMBER1*; *GLO: GLOBOSA; *MB: maximum likelihood bootstrap; ML: maximum likelihood; *PCF: PROLIFERATING CELL NUCLEAR ANTIGEN GENE-CONTROLLING ELEMENT BINDING FACTOR*; PP: Bayesian posterior probability; qRT-PCR: quantitative reverse transcriptase polymerase chain reaction; *RAD: RADIALIS; REP1: RETARDED PALEA 1; *SEM: SCANNING ELECTRON MICROSCOPY; SD: standard deviation; *SUP: SUPERMAN*; *TB1: teosinte branched1*.

## Competing interests

The authors declare that they have no competing interests.

## Authors' contributions

JCP designed and performed the experiments. JCP and LCH wrote the manuscript. All authors read and approved the final manuscript.

## Supplementary Material

Additional file 1 **Primers used for RT-PCR**. Primer sequences used for amplification of target and housekeeping genes from *Commelina communis, C. dianthifolia *and *Tradescantia pallida *are listed in table format. Click here for file

Additional file 2 **Second biological replicate for relative quantitative gene expression in dissected flower tissues**. In agreement with the first biological replicate (Figure 5), *GLO *expression is significantly higher in dorsal staminodes versus ventral stamens of *Commelina communis*, but is similar across inner tepals of both *Tradescantia pallida *and *C. communis *(left). *DEF *is again undetectable in ventral, but not dorsal, inner tepals of *C. communis*. By contrast, *DEF *expression is higher in ventral versus dorsal inner tepals of *Tradescantia pallida *(second from left). Similar to the first biological replicate, *TB1a *is undetectable in *T. pallida *inflorescence tissues, and is expressed significantly more strongly in both outer and inner ventral versus dorsal tepals of *C. communis *(second from right). *TB1b *is undetectable in inflorescence organs of *C. communis*, although the primers amplify genomic DNA, and is undetectable in inner tepals and gynoecia of *T. pallida *(right). *β-ACT *and *EF1a *were used as internal controls. Experiments were conducted with three to four technical replicates. Bars show mean and standard deviation. White bars, whole organs; blacks bars, dorsal organs; gray bars, ventral organs. Asterisks denote significant differences between dorsal and ventral organ comparisons. gDNA, genomic DNA; Inf, whole inflorescence; Dot, dorsal outer tepal; Vot, ventral outer tepal; Dit, dorsal inner tepal; Vit, ventral inner tepal; Dst, dorsal stamens/staminodes; VSt, ventral stamens; Gyn, gynoecia; nd, not detected. Click here for file

Additional file 3 **Best maximum likelihood phylogeny of monocot B-class genes**. Maximum likelihood bootstrap values above 69% (left) and Bayesian posterior probabilities above 89% (right) are shown for each branch. Asterisks denote 100% support. Dashes indicate bootstrap support below 70% only when the corresponding posterior probability is shown. The focal genes of this study are highlighted in gray boxes. Click here for file
