# Supplementary material for: Parallel evolution of TCP and B-class genes in Commelinaceae flower bilateral symmetry
Source: EvoDevo. 2012 Mar 6;3:6. doi: 10.1186/2041-9139-3-6 (PMC3359255; doi:10.1186/2041-9139-3-6)
Supplement: Additional file 1 — Primers used for RT-PCR. Primer sequences used for amplification of target and housekeeping genes from Commelina communis, C. dianthifolia and Tradescantia pallida are listed in table format. [file 2041-9139-3-6-S1.PDF]

Additional file 1 Primers used for RT-PCR

Name	Sequence (5'-3')	Genes amplified
Forward:		
CcACT62.F	CTTTCACCACCACAGCAGAA	*Cc*ß-*ACTIN*
CdACT.F	GGGCTGAGAGATTCAGATGC	*Cd*ß-*ACTIN*
TpEF1a-19-F	CACATCAACATTGTGGTCATTG	*TpEF1a*
CcDEF65qRT.F	AGAGGATGCAAAACCACCTG	*CcDEF, CdDEF*
CcGLO2qRT.F	TGCCTAAGATCTTGGAGAAG	*CcGLO*
CdGLO.F	ACTCTCTCCAACCCGAGGAT	*CdGLO*
TpDEF.F	TTCTGTGAAGGCTGTTCGTG	*TpDEF, TsDEF*
TpGLOa.F	GGATCATCAAGAAGGCCAGA	*TpGLO, TsGLO*
CcTB1.rt.F	TTCAGCCTTCAGGACTTGCT	*CcTB1a*
CcTB1b-232-F	ACCTCGGAGCATGAAGACAT	*CcTB1b*
CdTB1a.F	TCAGCCTTCAGGACTTGCTC	*CdTB1a*
CdTB1b.F	CAAGTCAAGAGGTGCCATCA	*CdTB1b*
TpTB1a-93-F	CAAGACATGCTTGGCTTTGA	*TpTB1a*
TpTB1b-157-F	GGTGCCATCAAAGAACTTCC	*TpTB1b*
Reverse:		
CcACT281R	GCTGCTTCCATGCCTATCAT	*Cc*ß-*ACTIN*
CdACT.R	GGTTGAGCCACCACTAAGGA	*Cd*ß-*ACTIN*
TpEF1a-241-R	CGAACTTCCACAGGGCAATA	*TpEF1a*
CcDEF291qRT.R	TGCTTCAGAGGAGTGCTTCA	*CcDEF,CdDEF*
CcGLO200qRT.R	ACGGATATGCGAAAGTCAGG	*CcGLO*
CdGLO.R	AAGCTCCAAGTCCCCAAACT	*CdGLO*
TpDEF.R	ATGAAGATTGGGCTGGTTTG	*TpDEF, TsDEF*
TpGLOa.R	TGCTTCTCGTCCCATAGCTT	*TpGLO, TsGLO*
CcTB1.rt.R	CTTAGCGCTTACCGACATCA	*CcTB1a*
CcTB1b-389-R	TTCGCCCTTGACTCTCTTGT	*CcTB1b*
CdTB1a.R	TTCGTACTCCGATGCTGATG	*CdTB1a*
CdTB1b.R	CGCGGTCTTAGTAGCAGCTT	*CdTB1b*
TpTB1a-335-R	ATGCAGTGGAGCATCTTTCA	*TpTB1a*
TpTB1b-405-R	TCTTGCTTTTGCCCTTGATT	*TpTB1b*
